# Method for Determining Treated Metal Surface Quality Using Computer Vision Technology

**DOI:** 10.3390/s22166223

**Published:** 2022-08-19

**Authors:** Anas M. Al-Oraiqat, Tetiana Smirnova, Oleksandr Drieiev, Oleksii Smirnov, Liudmyla Polishchuk, Sheroz Khan, Yassin M. Y. Hasan, Aladdein M. Amro, Hazim S. AlRawashdeh

**Affiliations:** 1 Department of Cyber Security, College of Engineering & Information Technology, Onaizah Colleges, Onaizah P.O. Box 5371, Saudi Arabia; 2 Department of Cybersecurity and Software, Central Ukrainian National Technical University, P.O. Box 25006 Kropyvnytskyi, Ukraine; 3 Department of Electrical Engineering, College of Engineering & Information Technology, Onaizah Colleges, Onaizah P.O. Box 5371, Saudi Arabia; 4 Department of Computer Engineering, Taibah University, Medina P.O. Box 2898, Saudi Arabia

**Keywords:** computer vision, processing methods, image segmentation, texture analysis, quantitative characterization, real-time analysis

## Abstract

Computer vision and image processing techniques have been extensively used in various fields and a wide range of applications, as well as recently in surface treatment to determine the quality of metal processing. Accordingly, digital image evaluation and processing are carried out to perform image segmentation, identification, and classification to ensure the quality of metal surfaces. In this work, a novel method is developed to effectively determine the quality of metal surface processing using computer vision techniques in real time, according to the average size of irregularities and caverns of captured metal surface images. The presented literature review focuses on classifying images into treated and untreated areas. The high computation burden to process a given image frame makes it unsuitable for real-time system applications. In addition, the considered current methods do not provide a quantitative assessment of the properties of the treated surfaces. The markup, processed, and untreated surfaces are explored based on the entropy criterion of information showing the randomness disorder of an already treated surface. However, the absence of an explicit indication of the magnitude of the irregularities carries a dependence on the lighting conditions, not allowing to explicitly specify such characteristics in the system. Moreover, due to the requirement of the mandatory use of specific area data, regarding the size of the cavities, the work is challenging in evaluating the average frequency of these cavities. Therefore, an algorithm is developed for finding the period of determining the quality of metal surface treatment, taking into account the porous matrix, and the complexities of calculating the surface tensor. Experimentally, the results of this work make it possible to effectively evaluate the quality of the treated surface, according to the criterion of the size of the resulting irregularities, with a frame processing time of 20 ms, closely meeting the real-time requirements.

## 1. Introduction

Images provide information on the biochemical composition, plant biology, and cytoskeleton of the target material sample, and surface fabrications of micro-machining industry. Images are segmented and evaluated to estimate useful statistical indices to extract them as recognizable features of interest through using computer vision technology. In computer vision techniques, a digital image is evaluated and processed through image segmentation for identification of recognizable features, or classification with respect to the surface texture to estimate quantitatively the unevenness or irregularities accordingly. The surface texture manipulation coupled with mathematical models for images produces a meaningful characterization of the target surfaces, indicating that the texture or surface analysis is valuable for distinguishing common surfaces from those with infection or treated surfaces from untreated surfaces. The texture features (TF) analysis in terms of the density distribution in the neighboring pixels was used to characterize crop diseases such as identification of yellow rust on wheat leaves [1,2] in terms of the comparison results using support vector machine (SVM) based on different features. Texture analysis was used to characterize cardiac tissues by quantification of gadolinium enhancement (LGE) with cardiac magnetic resonance imaging (MRI) to establish an association between some medical conditions of patients with hypertrophic cardiomyopathy (HCM) by studying intra-myocardial patterns in cardiac muscles [3]. The emphasis was on the use of machine learning (ML), showing such close similarities in terms of its applications in the case of either surface or cellular structure analysis.

The deep convolutional neural network (DCNN) has been fused with a segmentation-based fractal texture analysis to classify images obtained by converting malware binary code into grayscale images. The DCNN is optimized by comparing features extracted from a reference image with features of target images for the degree of similarity drawn using the proposed malicious malware classification model [4]. The CNN model has been used to classify skin for potential diseases using damaged versus healthy tissue features. The resulting computer vision technique performance is evaluated on images of skin affected by the varying level of diseases such as acne, a skin condition characterized by red pimples, keratosis, showing horny overgrowth on the skin, eczema herpeticum, a medical condition in which patches of skin become rough and inflamed with blisters that cause itching and bleeding, and urticarial causing red welts on the skin that cause intense itching; all such applications use datasets obtained from the DermNet [5]. Computer vision has been developing intelligent systems to analyze images obtained by UAV cameras in early fire protection by exploiting the dynamic textures of fires [6]. The deep convolutional neural networks (DCNN) approach has been equally used to parse the texture of cracks in the infrastructure by analyzing the images in terms of statistical indices [7]. The quantitative assessment of surface irregularities is of importance from Industry 4.0-related viewpoint applications. The proposed method is evaluated by comparisons made with previously reported DCNN-based models for crack detection accuracy and precision. In another approach, the current neural pattern transfer validation distillation methods are used for ultra-resolution of collaborative distillation for liquid mixture separation [8,9] to demonstrate the utility of the CNN approaches in terms of simplicity and feasibility of pattern transfer applications. The same practices may prove helpful to recognize micro-defects on irregular metal surfaces using photographs with specified properties.

The rest of the article is organized as consisting of related work that provides a critical review to formulate the core component of research dedicated to identical approaches to digital image processing using computer vision techniques. The next section describes the method used to determine the surface treatment quality of metals using computer vision techniques. The image-processing stages are illustrated and supported by related algorithm illustrations coupled with pseudocode. The problem is to find a set of suitable algorithmic steps to compute the differentiating features for distinguishing the image area distinctively. Moreover, the statistical properties of the image fragments, from the point of view of their suitability, are considered for distinguishing the treated surface areas from untreated metal surface. The necessity of developing a method related to the measured characteristics of the metal surface is worthy of intense attention. The development of a so-called algorithm to find the period of dominant oscillation contains the formulation of a mathematical derivation to obtain the mean period of difference in brightness, representing the average size of the cavities. The mathematical proof was constructed for the method for obtaining the average volume of cavities. It shows the applicability of the obtained characteristics, the average size of the cavities to characterize the image. The results appear in the conclusion of the article.

The main objective of this article is to propose a method for digital analysis of metal surface images that are processed in order to a mark an already processed metal surface for results having utility from an Industry 4.0-related applications viewpoint instead of pure metallurgical objectives. The main aim of the article is to extract the measure of irregularities in metal surfaces. The method was tested in terms of comparisons for each fragment using the Perlin gradient noise pattern, as expressed in Equation (9), to estimate the cyclic frequency in images for visual appearances.

## 2. Related Works

The task of characterizing images requires a critical review of relevant contemporary literature works. Since the technical images obtained during the processing of the metal surfaces have information-embedded structure, similar to any other bitmap images, the utility of these images is significant in terms of applicability to the problem in focus.

The CNN architecture has been used to process both high-resolution and low-resolution images to estimate severity of crowds exceeding scramble production threshold levels [10]. Image processing from MRI images has been used to study patients with hypertrophic cardiomyopathy (HCM) for different distribution patterns of cardiac pathology [11]. However, the vision-based techniques are usually used as a post-operative monitoring method, and thus lack the information embedded in the dynamic characteristics that are required in real-time monitoring. Thus, the surface texture analysis of high-resolution images may be useful in establishing a near-to-real-time monitoring system. The recognized text images of surface characteristics are detected using a CNN-based detection model that has already been trained on a similar dataset to detect fitting curved surfaces. The curve fitting process for the curved metal surface represents the appropriate curve in the case of the single-line text as well as multi-line text scenarios [12]. Image processing coupled with computer vision has been used to assess the machine surface, and thus indirectly the abnormal conditions of machine tools’ chatter vibrations and tool wear [13]. Texture analysis is based on the features of the evenness and roughness of the metallic surfaces in the area of surfaces in the field of detection and localization. Extensive quantitative experiments on real-world data show that the CNN-based classifier is superior to the latest approaches using feature extraction methods. The authors in [14] demonstrate a promising new application of deep neural networks to automate electrical discharge machining processes by sinking the mold into the image samples that show EDM steel surfaces with varying roughness. The surface roughness can be used to monitor the conditions of machine tools. The manufacturing tools are linked to sensors that collect signals about temperature, surface roughness, acoustic emissions, and vibrations through the Internet of Things (IoT) and cloud computing in the emerging Industry 4.0. The newly developed concepts of deep learning methods have achieved revolutionary success in light of the appropriate volume of data, compared to the classic artificial intelligence methods, toward realizing emerging developments that support the IoT [15]. The network-based VGG model for surface defects classification and detection of steel surface cavities or cracks has been addressed through traditional image processing techniques. Through experiments, compared to the original network, the improved algorithmic work is more effective in extracting features by showing higher-accuracy detection phenomena [16]. The literature review clarifies that original concept contribution has been so connected for maintaining the overall flow, keeping in mind that the equivalent extraction of cavity sizes related features are delivered. Deep learning, in conjunction with ultrasonic testing, is used for the porosity evaluation of additively manufactured parts designed through 3D computer-aided design procedures and was investigated for rough surfaces in [17]. The authors in [18] investigated the effectiveness by estimating roughness of surfaces produced as a result of the three methods of shot blasting, water jet texturing, and laser texturing. An automated visual inspection (AVI) tool targeting surface quality appears as a standard configuration for flat steel mills to improve product quality and enhance production efficiency, and was explored in [19,20]. The effect of the augmentation method was studied by adding fused spatial and channel attention modules to the network structure in the form of twin attention mechanism and relative mean (TARGAN) in [21,22], with an accuracy higher than the model, and to detect mild and severe imprint defects in stamped metal parts, a critical task for the automotive industry. The bottleneck of deep learning models is their training time, which is the main hindrance in the case that experiments in new images needing new training cycles are repeated. This issue is tackled by the authors in [23] where some weights are collected from training cycles along with some experimental parameters to develop a comprehensive assessment score correlated to the F2-score of detection models.

Thus, the task of developing a method for determining the quality of treated surfaces from untreated metal surfaces using computer vision technologies for real-time applications is justified. Table 1 summarizes the above-related research works related to texture or surface analyses to determine the characterizing features of surface conditions.

Based on the results of the literature review and related work, it follows that in modern systems of labeling images, the methods used are as summarized in Table 2.

Therefore, the fourth method is adopted in this work, which can avoid the large amount of equipment-specific workplace noise in the original image. Further, the main problem is the development with less computation power requirement of the calculated parameter that reflects the physical property in the metal surface digital image.

## 3. Determining the Quality of Metal Surface Treatment Using Computer Vision Technologies

**Figure 1:**
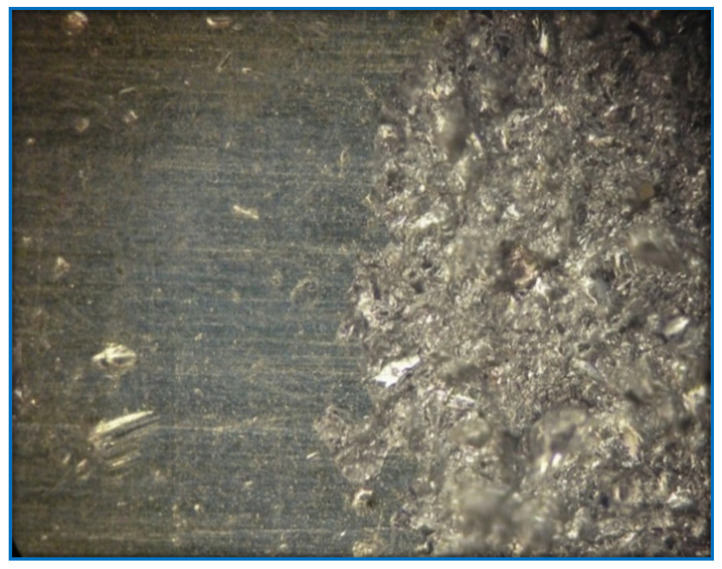
Partially treated sample of the metal surface by treating to increase the contact area of the base materials for spraying before “shot blasting”.

The specific task in the work is related to the hardening of the surface of the parts through selective processing. Most materials have insufficient adhesion strength to the substance on which they are sprayed. To increase the adhesion, shot blasting is used [18]. As a result of installation, irregularities are formed. These irregularities significantly increase the bonding area of the material substrate, and the spray. An example of a digital image of a partially treated surface is as shown in Figure 1, where the left side of the surface is not yet formed. The frame was captured with a video camera and video recorder that supports the Wide Quad Extended Graphics Array (WQXGA) format.

The ultimate task is to determine the average size of cavities on the provided image samples by focusing on the mathematical formulation of the problem, abstracting from the source of the image and the material used. As described above, we had to take up the use of neural networks to highlight the untreated portion of the surface. In this regard, it was necessary to take into account the basic properties of texture of the image fragment as:

1. Average brightness of the fragment.
2. Moment of distribution of brightness over a part of the image (dispersion).
3. Asymmetry.
4. Entropy of the information representing the image area, and similar indicators by image filtering the image.

**Figure 2:**
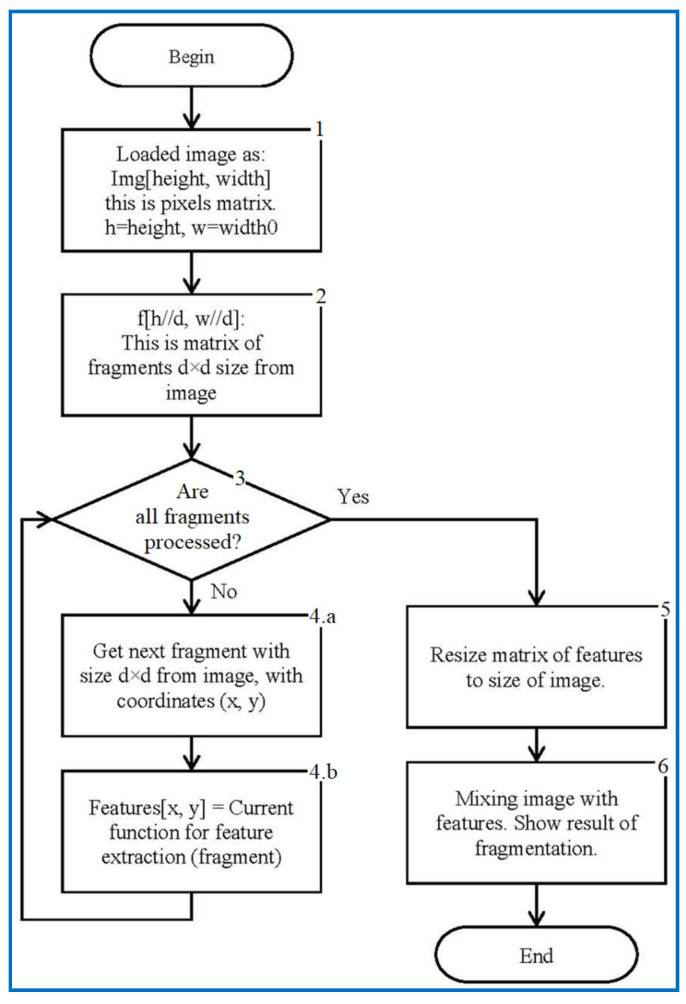
Flowchart for marking the image by texture properties.

The main image is broken into fragments of the same size. For each fragment, an arithmetic computation of the properties of the texture is made. Then, for each of the properties, a density map is obtained as an indicator of one or another texture property. The combination of the parameter values of the texture properties determines the nature of the texture in the selected part of the main image. The article explains the application of computer vision technique to assess the quality of metal surface in term of the frequency parameter, as in Equation (7). The relevance in terms of brightness diffusion on the input images was somehow addressed by authors in [24,25] to distinguish and highlight surface defects. The algorithm used in this work is as shown in Figure 2.

**Figure 3:**
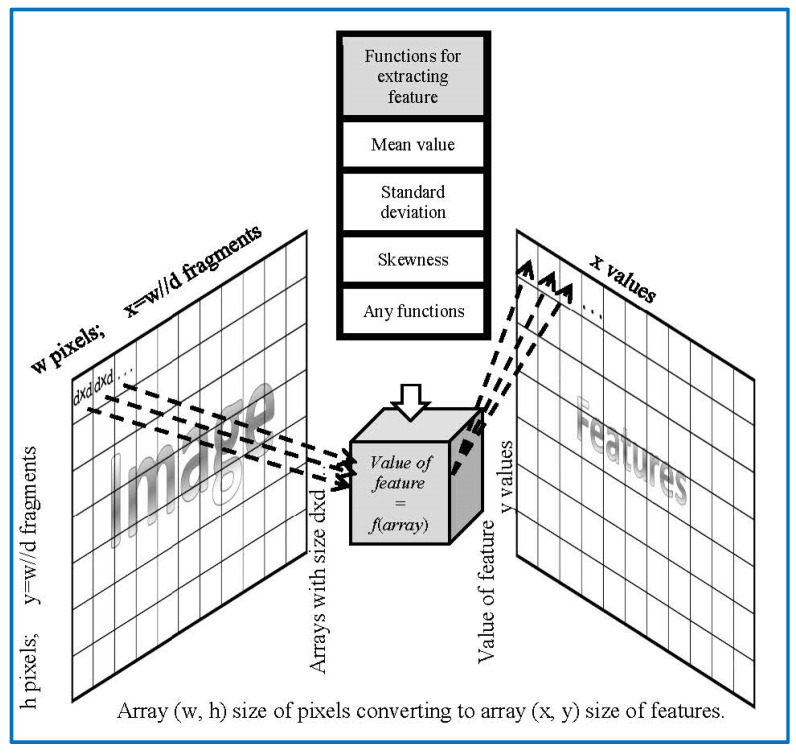
Functional scheme for image extraction features by algorithm of Figure 2.

**Figure 4:**
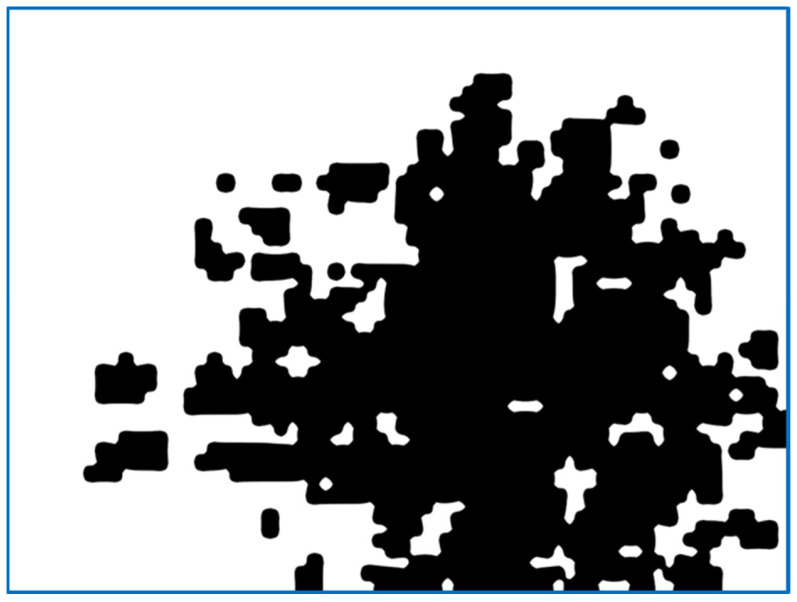
The feature selection result is based on the fragment average brightness or arithmetic mean of the brightness, 64 × 64 in size, with a step of 32 pixels.

**Figure 5:**
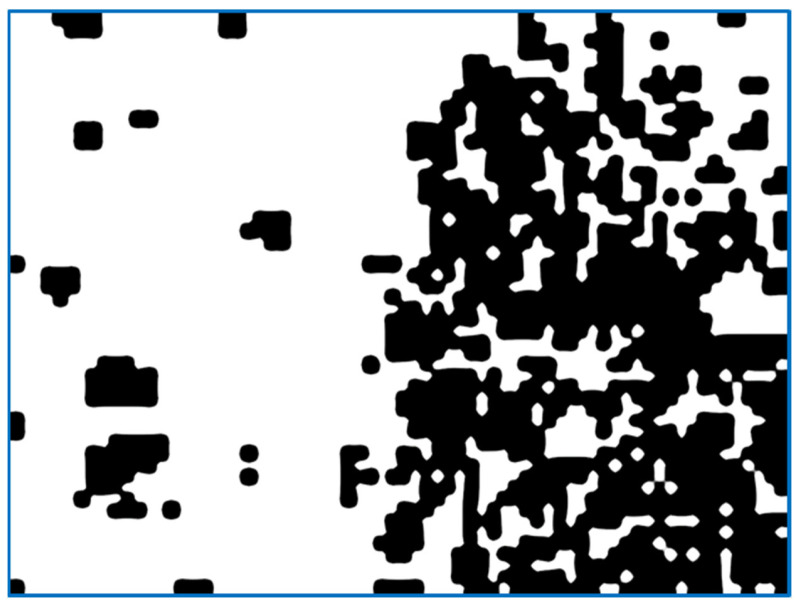
Dispersion feature result is based on brightness of 64 × 64 in size, with 32 pixels step.

**Figure 6:**
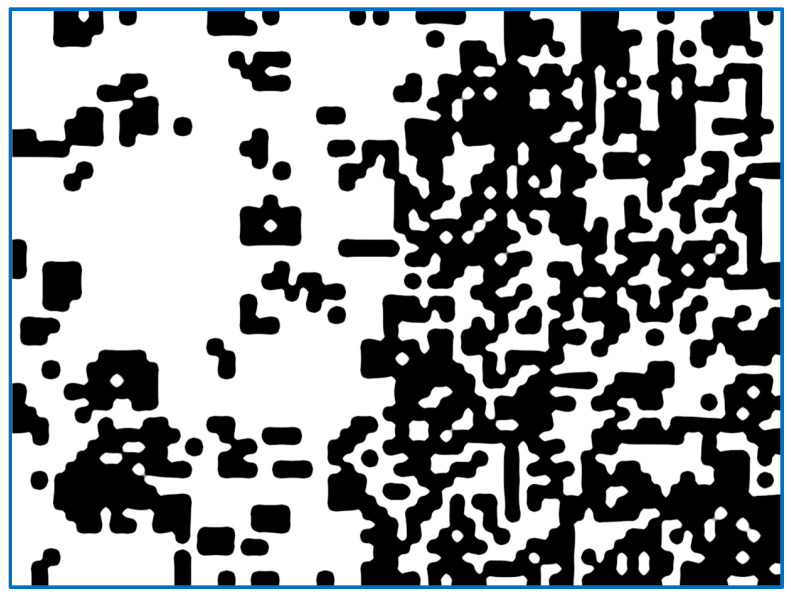
The asymmetry feature based on the brightness of a 64 × 64 fragment with 32 pixels step.

1. The whole image is read into the computer RAM as consisting of an array of pixels, made of ***h*** rows and ***w*** columns. Each pixel is represented in the range 0 to 255 according to brightness intensity.
2. The image is divided into square fragments with side “d” pixels. These fragments are stored in an array table f [h/d, w/d], as explained in Figure 2. The size of the table is “d” times smaller, both vertically and horizontally (matrix of fragments with a width, w//d, and a height, h//d, where “//” denotes division with integer answer).
3. The next block is to test the fragments that are taken in sequence from the table f, and a downward transition occurs after each of the fragments are identified by coordinates (x, y). When all fragments are processed, the loop exits to the right.
4. In the body of the loop, each fragment falls into a preselected function, which, based on the provided single fragment, calculates the property of this fragment. A few of the possible functions are discussed in the article. The result is added to the features table of fragment properties. The location of the calculated properties in the table coincides with the placement of the fragments themselves in the table f.
  (a) We send the image fragment into the function that gives the number indicating one of the properties of the texture represented by this fragment. The function is one of the implemented means applied to compute the value of the texture property. In this article, we develop mathematical equations to calculate average frequency, given in Equation (9), to segment the image.
  (b) The resulting value is entered into the [x, y] feature matrix. An example of the resulting matrix can be seen in Figure 3, Figure 4, Figure 5 and Figure 6 (for different features), where the target values of interest are shown by the change in brightness.
5. When exiting the loop, the resulting table of features is interpolated to the size of the input image (increased by d times). After normalizing the feature values with brightness ranging from 0 to 255, we obtain an image of the initial image fragmentation, according to the selected feature (as in Figure 3).
6. To improve the visibility of the result, the resulting fragmentation is mixed with the original image for showing features’ results clearly.

## 4. Texture Properties of a Fragment of an Image

For the segmentation of the image texture, the image is divided into parts, in which conditionally it is assumed that the properties of the image contained in this part appear uniformly. The existing statistical methods are used for highlighting the statistical parameters of the brightness distribution in these fragments. These properties are based on the characteristics of statistics and information. It is necessary to consider the applicability of the statistical features to the image encoding. Although these methods may not comply with the technical requirements, the effectiveness of their application will show the fundamental potential for solving the problem. The use of statistical properties, which satisfactorily allows marking of the treated part of the metal surface, allows us to design our method that will be sensitive to these statistical properties. 

If the arithmetic mean of the average brightness of a fragment is to be computed in the algorithm of Figure 2, we obtain the diagram as shown in Figure 4. It shows the average brightness while removing all other undesirable interference. The integrated brightness will be useful in the case of identical shooting conditions, such as a high-quality camera, as its brightness will be an essential feature of the texture.

A similar analysis, instead of the arithmetic mean of brightness, is the standard deviation that uses brightness of pixels in statistics analytical dispersion. It shows a lower sensitivity of texture as an indicator that accurately reflects the relative number of pixels, which differ from the average brightness. Therefore, it is more accurate to use the dispersion index to distinguish the textures consisting of approximately the same number of bright and dark pixels, referred to as banding. As shown in Figure 5, it is indeed a more uneven surface, more saturated with bright and dark pixels, giving dark spots in areas of the already treated surface. However, the accuracy of these markings is low and the absolute numbers will depend on the total illumination. Compulsory histogram expansion, that is, normalization of the original image, will also not remove the dependence on lighting. It should be noted that the value of the selected feature value appears as white if it exceeds the set threshold, otherwise it remains black.

The third statistical property is the moment of the distribution of brightness that gives the map as shown in Figure 6. This indicator interacts with an unequal number of bright and dark pixels, relative to the average value: a violation of the symmetry of the distribution in terms of brightness. It is normal to expect this number to rise on uneven surfaces. However, the question remains about the extent of these irregularities. In addition, this drawback is inherent in the above indicators. The skewness of the indicator will be effective when looking for striped or treated areas where the lines have significantly different widths. The value of the selected feature appears in white provided that it exceeds the set threshold limit, otherwise it remains black.

**Figure 7:**
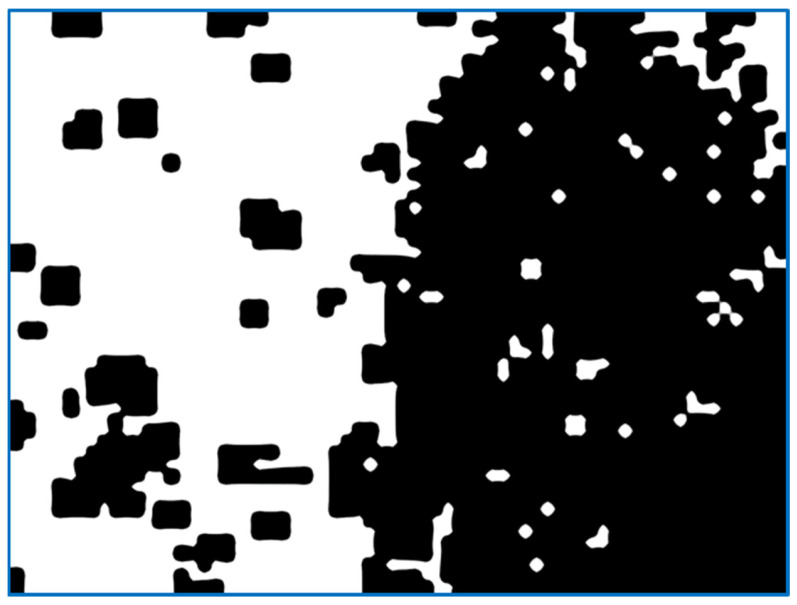
The result of feature extraction is obtained by computing the entropy of information, which represents the brightness distribution over a 64 × 64 fragment with 32 pixels step.

The following indicator is based on the concept of information entropy [19] when each fragment of the image of the processed surface is represented as a set of bytes, for which the entropy is calculated. Figure 7 shows the encoding obtained according to the information entropy value for each fragment. The higher value of entropy very well defines the areas of the image with an already treated surface. However, of all the methods described here, entropy is calculated using algorithms. It is more computationally intensive. The more significant drawback remains the unknown dependence of the irregularities and the value of information entropy of the image region. The entropy makes it possible to identify areas of interest with a reference to the measurable quality according to selected technical requirement, irrespective of the thresholding dictation.

The examples are considered to give an affirmative answer for the image distinguishability in the processed and unprocessed areas. However, the specific requirements for solving an engineering problem, according to an assessment of average magnitude of irregularities, remain unanswered. Accordingly, the lack of rapid methods for estimating the magnitude of irregularities justifies the task of developing our method.

## 5. Development of Algorithm for Finding Period Dominating Fluctuations

In the process of estimating the average magnitude of the irregularities, the development of the model is assumed wherein the irregularities are represented as complex harmonic oscillations with amplitude “*A*” and phase “*φ*” undergoing constant random changes. Therefore, to ignore the amplitude and phase of the oscillations, the authors decided to use local information near a single pixel wherein brightness is denoted as *p*, using the differential calculus, which is more applicable to lattice discrete space versus the differential calculus. In this way the average cyclic oscillation frequency, *ω*, is found that will give information about the magnitude of the surface roughness examined according to its digital image.

Assume the subscript “*i*” number is used to indicate the brightness of the pixels located sequentially. To search for the period of the dominant oscillation on three consecutive pixels, the difference, Equation (1), is used to seek the coefficient *a*:

(1) $$p_{i}+a\cdot p_{i+1}+p_{i+2}=0$$

The solutions of which are oscillations around the zero center of equilibrium at the moment of time *t,* as given in Equation (2):

(2) $$p_{i}=A\cos(\omega (t+i)+\phi )$$

Substituting Equation (2) into Equation (1) gives:

$$A\cos(\omega t+\phi )+a\cdot A\cos(\omega (t+1)+\phi )+p_{i+2}A\cos(\omega (t+2)+\phi )=0$$

cos(ωt+ϕ)+a⋅cos(ωt+ω+ϕ)+cos(ωt+2ω+ϕ)=0

To simplify further transformations, we use the complex exponent to switch from trigonometric to exponential functions, using the function of extracting the real part of the complex number and taking advantage of the properties of exponential functions to ultimately derive the simplified expression:

Re(ejωtejϕ(1+a⋅ejω+(ejω)2))=0

where “*j*” is the root of −1.

Then, we return to the trigonometric representation and take some further derivations in order to finally arrive at:

(3) Re((cos(ωt+ϕ)+jsin(ωt+ϕ))(cos(ω)+jsin(ω)))(a+2cos(ω))=0

To fulfill the condition of Equation (3), one of the factors must always be equal to zero. The Re part on the left is highlighting the real part, and depends on time and cannot always be equal to zero. Therefore, the right factor of Equation (3) is definitely zero, as

(4) a+2cos(ω)=0

Equation (4) for the image can be easily converted into deriving the cyclic frequency of *ω*, as in Equation (5):

(5) ω=arccos(−a/2)

However, the problem is that the photographic image has a plane dimension. However, when deriving Equation (5), only one dimension is used. Therefore, it is proposed to combine the vertical and horizontal axes according to the following:

$$\left(\begin{array}{ccc} 0 & 0 & 0\\ 1 & a & 1\\ 0 & 0 & 0 \end{array}\right)+\left(\begin{array}{ccc} 0 & 1 & 0\\ 0 & a & 0\\ 0 & 1 & 0 \end{array}\right)=\left(\begin{array}{ccc} 0 & 1 & 0\\ 1 & 2a & 1\\ 0 & 1 & 0 \end{array}\right)$$

Here, the matrices denote the filters that are applied to the bitmap. For the matrix that represents the filter, the element product is carried out with the corresponding pixels of the image $u_{x,y}$ after the central pixel is replaced by the sum of the formed $\Delta_{x,y}$ products. The result of actions can be shown by the following Equation (6):

(6) $$\Delta_{x,y}=u_{x-1,y}+u_{x+1,y}+u_{x,y-1}+u_{x,y+1}+2au_{x,y}$$

If we apply Equation (6) to the entire image, we obtain the full result of applying the filter, which in the ideal case should give zero, as in Equation (7):

(7) $$\sum_{x,y=1}^{N-2}{\left(\Delta_{x,y}\right)}^{2}=\sum_{x,y=1}^{N-2}{\left(u_{x-1,y}+u_{x+1,y}+u_{x,y-1}+u_{x,y+1}+2au_{x,y}\right)}^{2}$$

This makes it possible to use the least-squares method to find coefficient “*a*”, which will express the desired filter. For the found “*a*”, the filter will give the minimum deviation:

$$S=\min\left(\sum_{x,y=1}^{N-2}\left(\begin{array}{l} u_{x-1,y}^{2}+u_{x+1,y}^{2}+u_{x,y-1}^{2}+u_{x,y+1}^{2}+4a^{2}u_{x,y}^{2}+\\ +2u_{x-1,y}u_{x+1,y}+2u_{x-1,y}u_{x,y-1}+2u_{x-1,y}u_{x,y+1}+4au_{x-1,y}u_{x,y}+\\ +2u_{x+1,y}u_{x,y-1}+2u_{x+1,y}u_{x,y-1}+2u_{x+1,y}u_{x,y+1}+\\ +4au_{x+1,y}u_{x,y}+2u_{x,y-1}u_{x,y+1}+4au_{x,y-1}u_{x,y}+4au_{x,y+1}u_{x,y} \end{array}\right)\right)$$

The minimum is sought through using the extremum point condition at which the derivative of the change in the sum of the squares of the deviation is equal to zero:

$$S{’}_{a}=\sum_{x,y=1}^{N-2}\left(8au_{x,y}^{2}+4u_{x-1,y}u_{x,y}+4u_{x+1,y}u_{x,y}+4u_{x,y-1}u_{x,y}+4u_{x,y+1}u_{x,y}\right)=0$$

It remains to solve the formulated equation:

$$2a\sum_{x,y=1}^{N-2}u_{x,y}^{2}+\sum_{x,y=1}^{N-2}u_{x,y}\left(u_{x-1,y}+u_{x+1,y}+u_{x,y-1}+u_{x,y+1}\right)=0$$

(8) $$a=-\frac{\sum_{x,y=1}^{N-2}u_{x,y}\left(u_{x-1,y}+u_{x+1,y}+u_{x,y-1}+u_{x,y+1}\right)}{2\sum_{x,y=1}^{N-2}u_{x,y}^{2}}$$

The Equation (9) is obtained by substituting Equation (8) into Equation (5):

(9) $$\omega =\arccos\left(\frac{\sum_{x,y=1}^{N-2}u_{x,y}\left(u_{x-1,y}+u_{x+1,y}+u_{x,y-1}+u_{x,y+1}\right)}{4\sum_{x,y=1}^{N-2}u_{x,y}^{2}}\right)$$

Equation (9) is easily implemented in the matrix and tensor computation libraries functions of TensorFlow or NumPy. Below is an example of a possible implementation of the predominant frequency finding function of Python code using the NumPy library with pseudocode reproduced, as given in Appendix A.

**Figure 8:**
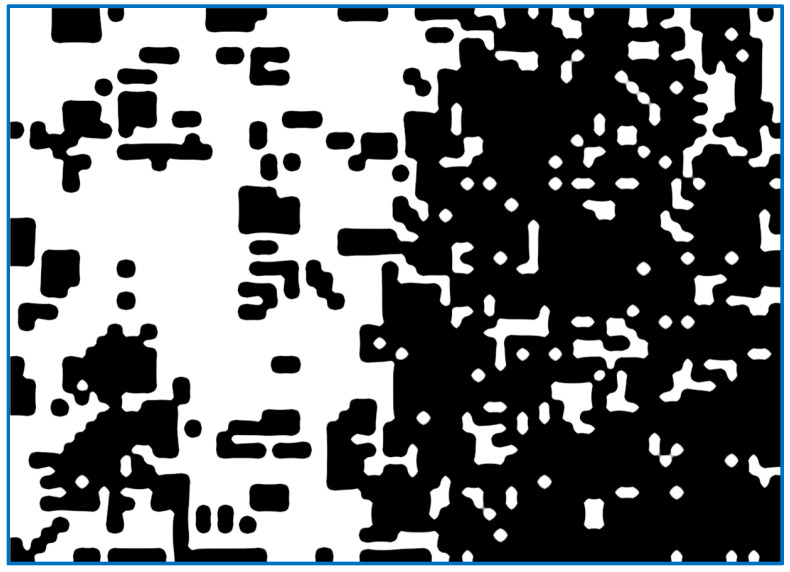
Result highlighting the frequency feature by brightness of a 64 × 64 fragment with 32 pixels step (Equation (9)).

Figure 8 shows similar to the above examples in Figure 4, Figure 5, Figure 6 and Figure 7; the quality of highlighting the black-treated surface is visually comparable to the entropy index. However, each fragment does not contain an abstract numerical indicator; instead, it contains a value associated with the amount of roughness that is to be marked.

The result of image segmentation is obtained, which is comparable in quality to that of the information entropy index of the image fragment. However, the result obtained does not involve computation of algorithms. Hence, the computation time is reduced significantly as it relies on the geometric dimensions of the cavities. Therefore, the segmentation results can be objectively compared with the measurement of the linear dimensions of the irregularities on the surface of the metal. As a result, the computations are faster in completing the technical task, which indicates a correlation with an independent physically measured parameter.

**Figure 9:**
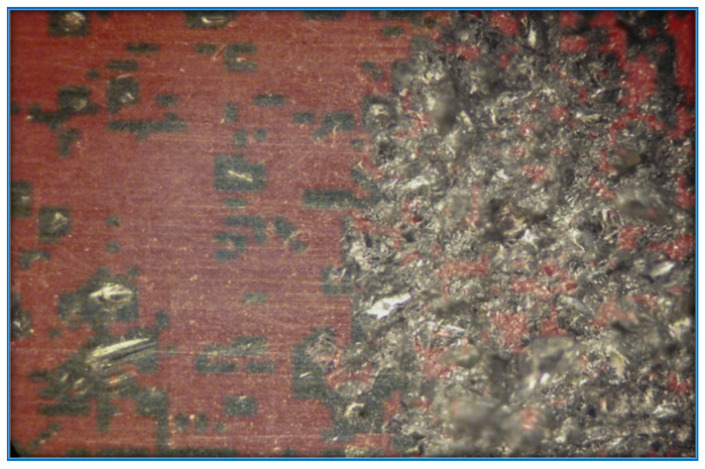
Marking of insufficiently processed surfaces, using the index, *ω*, in Equation (9).

The marking quality is visually shown in Figure 9. It is clear that the frequency index, while ignoring the general changes in illumination and contrast, clearly interacts with the presence of many small elements that arise with the riveted metal surface.

The cyclic frequency, expressed by Equation (9), allows us to estimate the oscillation period T = 2π/*ω*, which corresponds to the magnitude of the irregularities in pixels.

## 6. Experimental Details

**Figure 10:**
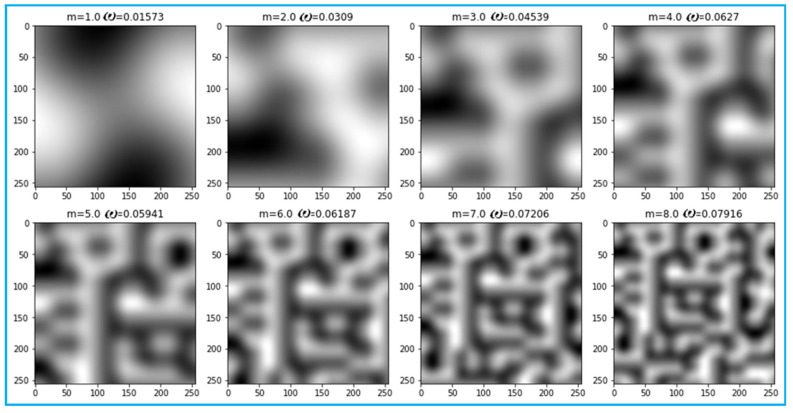
Perlin noise by zoom factor, m, and result analysis by Equation (9) of angular frequency, ω.

For determination of the accuracy of the method, the average size of irregularities requires a large number of field experiments. This is difficult to implement as it requires a lot of manual work. In the case of creating synthetic models for photographic images with irregularities, the number of experiments can be significantly increased. Periodic functions can be taken as a texture of the experiment to determine the average size of image elements, but the real picture is random. Therefore, to increase the acceptance realism, it was decided to use the random Perlin noise. It bears a strong resemblance to real noise, as shown in Figure 10. It contains a scaling factor, m, which shows the average number of cycles of brightness changes in a fragment of the figure. All fragments have axes on which the ordinal numbers of pixels are marked, vertically and horizontally.

In addition, for each fragment of the Perlin noise pattern, using Equation (9), an estimate of the cyclic frequency, *ω*, is obtained. Since the scaling is unknown, it suffices to show that there is a linear relationship between the quantities of scaling factor, m, and the cyclic frequency, *ω*. The random nature of Perlin noise introduces random errors into measurements according to Equation (9). Therefore, to increase the reliability of the results, the experiment for obtaining the pairs of values (*m*, *ω*) is repeated several times. For the number of experiments, *n* > 50, the error, *μ*, of the correlation coefficient, *r*, is determined by the formula $\mu =(1-r^{2})/\sqrt{n}$, and then the real correlation coefficient will be within the range of *r* ± 3*μ*. 

The results of 1000 experiments gave the following result:

$$r=\frac{\sum_{i=1}^{n}\left(m_{i}-\bar{m})\cdot (\omega_{i}-\bar{\omega }\right)}{\sqrt{\sum_{i=1}^{n}{\left(m_{i}-\bar{m}\right)}^{2}\cdot \sum_{i=1}^{n}{\left(\omega_{i}-\bar{\omega }\right)}^{2}}}\approx 0.9576$$

$$3\mu =3(1-0.9576^{2})/\sqrt{1000}\approx 0.0079$$

where the line above means the arithmetic mean (measured mathematical expectation) of the values obtained. As a result, *r* = 0.96 ± 0.008; the presence of a linear relationship between the scale factor and estimate of *ω* is confirmed.

**Figure 11:**
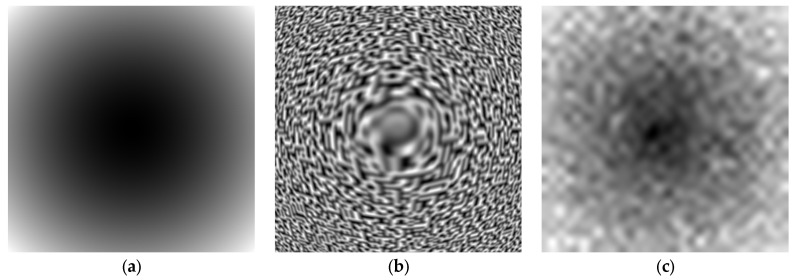
Demonstration of zoom factor reconstruction based on Perlin noise. (**a**) Frequency diagram etalon. (**b**) Perlin noise. (**c**) Restored frequency diagram.

For clarity, the values of “*ω*” are measured by Perlin noise, where the size of the elements changes with distance from the center of Figure 11.

For clarity, the change in the scale of the oscillations is shown in a single image. The processing is the same for the image in Figure 11. In our approach, we used a synthesized image as a test, in which the image of the treated metal surface was simulated by Perlin noise and is shown in Figure 11b in order to better represent the complexity in visual effects for the range in frequency shown in Figure 11a. It may be noted that Figure 11a shows the etalon in the wavelength of light interference. However, visual comparison is complicated by the nonlinear nature of human perception. Therefore, the given parameter in Figure 11a is represented by its equivalent frequency diagram, as shown in Figure 11c, using statistical methods.

The restored frequency plot of Figure 11c is obtained from image Figure 11b by substituting 64 × 64 pixel image fragments into Equation (9). The fragment size is selected according to the expected size of the elements in the image being analyzed.

In addition, since the absolute error changes a little bit in the measured range, the relative error reaches 100% for irregularities, the size of which significantly exceeds the size of the fragment that is being analyzed and decreases with the size of the irregularities. This makes it possible to develop in our further work an automatic selection of the size of the fragments in order to measure the dimensions of irregularities, based on the knowledge of the expected parameters of the metal surface before and after processing. The article presented the use of computer vision for applications of metal surface quality assessment. The utility of this work lies in the appropriate tuning of the frequency as a requirement for a particular application [26]. The technique can be usefully applied for localization of rusts using RGB images on metal structures [27] or corrosion detection [28], and possibly a close resemblance to the work in [29,30].

## 7. Conclusions

In this article, a method was developed to determine the quality of metal surface treatment using computer vision techniques. The results may prove useful for real-time applications. The quality was determined according to the average size of the irregularities of cavities. A review of the literature has demonstrated the availability of methods for distinguishing images in treated and untreated surface areas with qualitative details; however, the current methods do not provide quantitative assessment. In this work, experiments were conducted using common texture properties for converting intensity of pixels into an equivalent frequency variable. The technique used ensures that each fragment of the image is transformed into frequency variable as equivalent of brightness intensity. Further, the algorithm was implemented such that each fragment is recognized relative to the brightness of the central fragment, making the image a whole single commodity. This demonstrated the conditional applicability of labeling for treated and untreated surfaces, based on an information entropy criterion that grows for an already treated surface. However, the lack of an explicit indication in terms of the irregularities as illumination (contrast)-specific-dependent magnitude do not allow the use of these characteristics to be displayed explicitly. The execution of computed algorithms significantly increases the computation time. The frame was processed for almost 5 s in the best computation (Figure 6 results), which is not suitable for real-time systems. In connection with the requirements for linking the decision-making to the measured parameter, the work solved the problem of a local estimate of the volume obtained during the processing of irregularities. As a result, an algorithm was developed for finding the period of the dominant oscillations using the advantages of the matrix and tensor computation libraries functions. This allowed an objective assessment of the quality of the surface treatment according to the size of the irregularities (cavities). While using the proposed method, the results of Figure 7 show that using the Intel i7 processor family 9000 series, WQXGA frame computation takes 20 millisecond, encouraging features to promise for real-time application requirement. As a result of numerical modeling of synthesized Perlin noise in terms of variable frequency, it was shown that the obtained method reflects the properties of brightness contrast in the image area of the average size. The proposed method developed is applicable for practical usage both for local systems and the Internet service modules. The results differ from similar ones in that the marking of the treated surface moves from a subjective assessment to an objective assessment with the possibility of checking the result using linear measuring devices in real-time systems. In this article, we developed mathematical formulae that give an output value proportional to the size of the “wave” in the input image by computing the average frequency to image segment according to the compositional features in need, proving *r* = 0.96 ± 0.008 as a linear relationship between the scale factor, *m*, and the estimate of *ω* is confirmed. The utility of this work finds application in the IoT-enabled environment in Industry 4.0 in the field of micromachining related to flexible electronics. However, for real-time application there is a need to find the balance of algorithm complexity, detection accuracy, and detection time [23].

## Figures and Tables

**Table 1:** Approaches comparison.

Source	Approach Used	Target	Data Type	Restrictions
[1]	Clustering, based on the vector of statistical features and features, based on information properties and filtering (1042 features in total), and followed by the elimination of uninformative features.	By the properties of the selected segments, determine potentially severe cases, the course of the disease.	Images obtained from computed tomography.	Initially, the selected signs are not associated with the studied phenomenon, but they are used according to the presence of correlation dependences. As a result, there is no explanation of the mechanism of the dependence of the result on the selected features.
[2]	Segmentation of the image, based on color gradation, improved by taking into account the statistical properties of the texture in the area of the image.	Identify the infected areas on the shoots.	Hyperspectral digital bitmap.	The method is based on the use of the spectral properties of chemical components of plant samples.
[3]	Statistical properties of a texture (a collection of pixels) on a portion of a digital image.	Highlight myocardial disorders.	Bitmap digital image.	For other applications, it is necessary to first investigate the dependence of the statistical properties of the texture and the significant indicators in a new task.
[4]	Clustering image features that are obtained from an already trained neural network (reducing the dimension of the feature vector).	Determine the presence of malicious code in the executable file.	Software byte code that is represented as a digital bitmap image in grayscale.	The method gives the probability of the presence of certain features in the image; however, there is no reasoning for the decision made. Requires a lot of training data.
[5]	Image markup using convolutional neural network.	Classify skin lesions.	Color, digital, raster, photographic image of a site of human skin.	The system can be used only for recommendations, due to the lack of argumentation, for the decisions made. Requires a lot of training data.
[6]	Image markup using a neural network.	Mark sources of ignition in images.	Color panoramic digital raster photographic image of the Earth’s surface areas obtained from low-flying vehicles.	Lack of argumentation for the decisions made, requires a lot of training data.
[7]	Marking images using a neural network.	The markings on the image, the roadbed damage.	Color, digital, raster, photographic image, roadbed.	Requires a lot of training data. Can pass mild damage, which is acceptable for this task.
[8]	Convolutional neural network, as part of the encoder–decoder.	Generation of a texture, with specified properties, based on a high-resolution sample.	Donor texture, a random vector of parameters, for a variety of results.	The system can be used to transfer a style or generate a family of textures with the same properties. One neural network is capable of generating only one texture.
[9]	A neural network for the extraction of texture features, with a gradient descent of the image to enhance the given texture features.	Image style transfer.	Donor digital image and resizable image.	Textural features are abstract, the significance of individual components, textural features are unknown. It is possible to use automatic segmentation based on the clustering of the received features. The belonging of the obtained texture classes requires a separate study in each case.
[10,11]	Crowd analysis to estimate crowds’ density and chest MRI images analysis for HCM diagnosis.	Crowd videos, images, and chest MRI.	Articles relate to producing digital images as color intensity variation.	Static conditions instead of dynamic.
[12,13,14,15,16,17,18,19]	Related to detection of curved surfaces features for tools conditions monitoring through data collection via varying sensing means.	Surfaces images.	Articles relate to obtaining images through various means.	The density estimation for estimating the target parameter of surface roughness or wear or chatter of the tools.
Our approach	Computation of relevant parameters reflecting the target surface image areas identifiably, and in linear relation to the metal surfaces irregularities.	Select areas of image irregularities have a specified range of illumination values.	A digital raster image, in grayscale, with a known scale.	Metal surface irregularities fit into the crop fragment of the image under study, as an example of a texture unit (the work uses image segmentation).

**Table 2:** Comparison table of segmentation methods.

#	Methodology	Principle of Operation	Advantages	Flaws
1	Highlighting by levels.	It is determined by highlighting zones of the same type, by color, and/or brightness (other criteria are possible). Analogs of geodetic lines are formed, which limit areas of the image.	Very fast.	Sensitive to noise, as in the corollary, not applicable to images with high detail.
2	Clustering.	Clustering techniques are used, such as K-means or any others. Clustering algorithms are applied not to the pixels of an image fragment, but their statistical (or other) properties.	Applicable to a wide class of images.	Requires significant computing resources.
3	Neural networks.	Trained neural networks are used (fully connected, convolutional, ResNet, transformers, or mixers).	Best markup quality indicators.	Requires a significant number of already segmented images to train.
4	Cutoff (threshold values).	For each fragment of the image, a computed parameter is determined. If this parameter is outside the specified range, the area of the image is discarded.	Performance depends on the complexity of computation, the cutoff parameter.	Requires scientific research for formalization (formulation, construction?), a parameter that will allow you to obtain the required markup.

## Data Availability

Not applicable.

## Appendix A

  ***Definition of Parameters and Legend:***

  ***W****—Width of image*

  ***H****—Height of image*

  ***img****—matrix of pixels of grayscale image from camera with resolution **w** (width) by **h** (height)*

  ***d****—Step of frequency analyzes, experimental define and depend by material and used camera; recommendation for select this value from 16 to 64 pixels*

  ***W****—Array of frequency value* ***ω*** *with resolution **w** (width) by **h** (height)*

[***x***,***y***]*–Coordinates of pixel positional, x–horizontal, y–vertical*

   ***ω****—Frequency value*

   ***//****—integer division*

   ***F****—Submatrix of image*

***Input*** *: —Grayscale image **img***

***Result*** *: Array W of frequency value ω*

*1: Find maximum value in image M = max (img)*

2: *Normalize image by all pixels img* [*x, y*] *= 2*img* [*x,y]/M-1. Now every pixel has lighting value from −1 to 1.*

3: *Initialize array of zero values W with size w//d by h//d*

4: *Traversal of all fragments in image with square window size d by d, with coordinates* [*i, j*]. *Let i range in* 0, 1, …, *w*//*d*, *let j range in* 0, 1, …, *h*//*d*

  4.1: *F is a submatrix of image img*[*i * d…i * d + d*, *j * d…j * d + d*] *width size d by d values*

  4.2: a = sum from x = 1 to d-2 {sum from y = 1 to d-2 {F [x, y] * F [x, y]} }

  4.3: b = sum from x = 1 to d-2 {sum from y = 1 to d-2 {F [x, y]*(F [x−1, y]+ F [x + 1, y] + F[x, y + 1] + F[x, y − 1])} }

  4.4: *ω =* arccos (b/(4 * a))

  4.5: W [i, j] = *ω*

5: *Resize matrix W to size* (*w*, *h*) *(with or without interpolation)*

It is very convenient to implement the algorithm using tabular calculations. Below is a fragment of the program code in Python using the capabilities of the NumPy library for release function for calculate frequency of submatrix (implement of 4.1–4.4 strings from pseudocode:

Import numpy as np

def get_frequency(img_gray: np.ndarray):

***i***mg_gray = img_gray-img_gray.min()

img_gray = img_gray/img_gray.max()

img_gray = img_gray-np.float32 (0.5)

       s4 = img_gray [0:−3, 1:−2] + img_gray [2:−1,1:−2] + \

       img_gray [1:−2, 0:−3] + img_gray [1:−2, 2:−1]

       p4 = np.multiply(s4, img_gray [1:−2, 1:−2])

       s = p4.sum()

       d4 = np.multiply(img_gray [1:−2,1:−2], img_gray [1:−2,1:−2])

       d = d4.sum()

       a = 0.25 * s/d

***ω*** = np.arccos (a)

return ***ω***
